# Characterization of novel *lncRNA* muscle expression profiles associated with meat quality in beef cattle

**DOI:** 10.1111/eva.13365

**Published:** 2022-03-25

**Authors:** Maria Malane Magalhães Muniz, Larissa Fernanda Simielli Fonseca, Daiane Cristina Becker Scalez, Aroa Suarez Vega, Danielly Beraldo dos Santos Silva, Jesus Aparecido Ferro, Artur Loyola Chardulo, Fernando Baldi, Angela Cánovas, Lucia Galvão de Albuquerque

**Affiliations:** ^1^ School of Agricultural and Veterinarian Sciences São Paulo State University (Unesp) Jaboticabal SP Brazil; ^2^ Department of Animal Biosciences Centre for Genetic Improvement of Livestock University of Guelph Guelph Ontario Canada; ^3^ 207340 National Council for Scientific and Technological Development (CNPq) Jaboticabal SP Brazil; ^4^ College of Veterinary and Animal Science São Paulo State University (Unesp) Botucatu SP Brazil

**Keywords:** genic lncRNA, lincRNA, marbling, RNA‐Seq, tenderness

## Abstract

The aim of this study was to identify novel lncRNA differentially expressed (DE) between divergent animals for beef tenderness and marbling traits in Nellore cattle. *Longissimus thoracis* muscle samples from the 20 most extreme bulls (of 80 bulls set) for tenderness, tender (*n* = 10) and tough (*n* = 10) groups, and marbling trait, high (*n* = 10) and low (*n* = 10) groups were used to perform transcriptomic analysis using RNA‐Sequencing. For tenderness, 29 lncRNA were DE (*p*‐value ≤ 0.01) in tough beef animals in relation to tender beef animals. We observed that genic lncRNAs, for example, *lncRNA_595*.*1*, were overlapping exonic part of the *PICK* gene, while *lncRNA_3097*.*2* and *lncRNA_3129*.*5* overlapped intronic part of the genes *GADL1* and *PSMD6*. The lncRNA associated with *PICK1*, *GADL1*, and *PMD6* genes were enriched in the pathways associated with the ionotropic glutamate receptor, gamma‐aminobutyric acid synthesis, and the ubiquitin–proteasome pathway. For marbling, 50 lncRNA were DE (*p*‐value ≤ 0.01) in high marbling group compared with low marbling animals. The genic lncRNAs, such as *lncRNA_3191*.*1*, were overlapped exonic part of the *ITGAL* gene, and the *lncRNA_512*.*1*, *lncRNA_3721*.*1*, and *lncRNA_41*.*4* overlapped intronic parts of the *KRAS* and *MASP1* genes. The *KRAS* and *ITGAL* genes were enriched in pathways associated with integrin signaling, which is involved in intracellular signals in response to the extracellular matrix, including cell form, mobility, and mediates progression through the cell cycle. In addition, the lincRNAs identified to marbling trait were associated with several genes related to calcium binding, muscle hypertrophy, skeletal muscle, lipase, and oxidative stress response pathways that seem to play a role important in the physiological processes related to meat quality. These findings bring new insights to better understand the biology mechanisms involved in the gene regulation of these traits, which will be valuable for a further investigation of the interactions between lncRNA and mRNAs, and of how these interactions may affect meat quality traits.

## 1 INTRODUCTION

Meat quality is defined by the product nutritional composition and by a group of subjective consumer perceptions as visual appearance, smell, firmness, juiciness, tenderness, and flavor (FAO, 2014; Maltin et al., 2003). Results from meat consumption studies indicate that quality is even more important than the product price to consumers (Henchion et al., 2014). Among the many factors that affect meat quality, the main one reported by consumers is tenderness (Fletcher, 2002), and another major factor is marbling, which affects both flavor and juiciness (Williams, 2008). These are complex traits, controlled by many genes and greatly influenced by environmental factors (Leal‐Gutiérrez & Mateescu, 2019). Moreover, these traits are expensive and difficult to measure, making the application of traditional selection, based‐phenotype and pedigree, even more difficult, since it requires the slaughter of the animals, leading to an increase in the generation interval and a decrease in genetic gain (Magalhães et al., 2016).

An approach that can collaborate to increase the knowledge about the genetic regulation of these traits is transcriptomics. The understanding of the individual genetic mechanisms behind transcript expression profiles involved in the variation of meat quality traits (e.g., tenderness and marbling) are still unclear, and further studies about regulatory elements which control gene expression of complex traits are necessary. The long noncoding RNA (lncRNAs) have a significant role in a wide variety of important biological processes, such as gene expression regulation and control of translation or genomic imprinting (Wucher et al., 2017). The lncRNAs are transcripts longer than 200 nucleotides and without any protein‐coding capabilities (Etebari et al., 2015; Gupta et al., 2019). Over the last decade, several lncRNAs have been identified and characterized, and this became possible because of the implementation of whole transcriptome sequencing technologies (Yao et al., 2019). However, few studies on the overall expression patterns of lncRNAs in *Longissimus thoracis* muscle have been conducted (Sun et al., 2016; Zhang et al., 2020), and they are even scarcer for meat quality traits. One of those few studies reported on this field was performed by Jiang et al. (2020) that investigated the transcriptome profiling of lncRNA related to fat tissues in Qinchuan cattle. Another study, by Zhang et al. (2019), investigated the molecular and expression characteristics of a novel lncRNA (*lncFAM200B*) along with its crucial genetic variations. They reported that the *lncFAM200B* expression trend is positively correlated with *MyoG* and *Myf5* expression in myoblast proliferation. However, no studies on lncRNA expression profile for meat tenderness in beef cattle have been reported. There is still much to discover about the lncRNA functions involved in cellular regulation pathways in meat quality traits. Thus, the objective of this study was to identify novel long genic and intergenic noncoding RNA, differentially expressed in *L. thoracis* muscle of Nellore cattle divergent for tenderness and marbling phenotypes. These results would provide new insights about the lncRNA function within a context of meat quality traits and enhance scientific data for further investigation in beef cattle breeding.

## 2 MATERIALS AND METHODS

### 2.1 Samples set and RNA‐seq data preparation

Animals were selected from a population of 80 Nellore bulls belonging to the Capivara farm located in Sao Paulo state, Brazil, which participates in the Nellore Qualitas breeding program and belonged to the same contemporary group (Muniz et al., 2021). Animals were slaughtered with an average age of 24 months, in a commercial slaughterhouse. Samples of the *L. thoracis* muscle were collected between the 12th and 13th ribs of the left half carcass. Two samples were obtained at two times: one sample at the time of slaughter, for RNA extraction, and another sample 24 h after slaughter for meat quality evaluation by methodologies described by Fonseca et al. (2017), Fonseca et al. (2020), Santos et al. (2020), and Muniz et al. (2020) that used the same dataset.

Tenderness was evaluated by Warner‐Bratzler shear force (WBSF), following the methodology proposed by Bratzler (1949), using the mechanical salter WBSF device. The visual marbling scores were evaluated according to USDA Quality Grade methodology (2000), which uses a marbling classification scale with values ranging from 1 to 9, being 1 value applied to absent marbling (devoid) and 9 corresponding to excessive marbling (moderately abundant). However, in Brazil, this scale ranges from zero (devoid) to six (moderate), because of the low degree of marbling in the Brazilian herd (Fonseca et al., 2020; Fonseca, Suárez‐Vega, et al., 2020).

According to the phenotypes scores obtained, the samples were sorted, and the 20 most extreme animals, for each trait, were chosen, composing two groups of 10 animals each (HIGH (*n* = 10) and LOW (*n* = 10) values) for marbling score and tenderness. The Student’s *t*‐test was used to verify the average difference between both groups. The low‐marbling grade groups had an average of 2.26 ± 0.05 and high‐marbling grade groups had an average of 3.26 ± 0.12. In addition, the tender meat (LOW group) had an average of 4.11 ± 0.30 (kgf), and the tough meat groups (HIGH group) had an average of 8.93 ± 1.23 (kgf). The groups were statistically different by the Student’s *t*‐test (*p*‐value < 0.001).

The RNA extraction was performed by methodologies described by Fonseca et al. (2017), Santos et al. (2020), and Muniz et al. (2020) that used the same dataset. The integrity of the RNA samples was assessed in the Agilent 2100 Bioanalyzer (Agilent, 2009), and the RNA concentration and genomic DNA contamination were determined using the Qubit^®^ 2.0 Fluorometer (Invitrogen, 2010) (Fonseca et al., 2017). RNA integrity number values were higher than 7.0 for all muscle samples, indicating good RNA quality. Paired end (2 × 100 bp) reads were sequenced using the HiSeq 2500 sequencer (Illumina).

### 2.2 RNA‐seq expression analyses

For the initial steps of the analysis, quality control of reads was performed based on Phred score, and GC content and over‐represented sequence parameters, using the CLC Genomics Workbench software 20.0.4 (CLC Bio), following the parameters described in Cánovas et al. (2014).

#### 2.2.1 Large gap mapping and transcript discovery

The “Large Gap Read Mapping” tool, implemented in CLC Genomics Workbench software 20.0.4 (CLC Bio), was used to map the paired‐end sequence reads according to the annotated reference genome ARS.UCD1.2 (ftp://ftp.ensembl.org/pub/release‐95/genbank/bos_taurus/) following the parameters: length fraction and similarity fraction = 0.8; two mismatches; and three insertions and three deletions per read were allowed. The Large Gap Read Mapping tool maps reads to a reference, allowing for large gaps in the mapping. It is developed to support transcript discovery using RNA‐seq data, since it is able to map RNA‐seq reads that span introns without requiring prior transcript annotations, for more details see Muniz et al. (2021).

CLC Genomic workbench's Transcript Discovery plugin (CLC Bio; Release 20.4) was used for transcript discovery in bovine based on genome reference (ARS.UCD1.2; ftp.ensembl.org/pub/release‐100/fasta/bos_taurus/). This tool has an algorithm that takes large gap read mapped as input, here each trait was analyzed separately, tenderness and marbling phenotypes (i.e., one group of high and other group of low for each trait). For reads mapping, the following criteria were allowed: mismatch (2), insertion (3), and deletion (3) costs; were allowed a minimum similarity (0.8) and length fraction (0.9) between a mapped segment and the reference; and a gap with maximum 50 Kbp distance between mapped read segments to span the introns from RNA‐Seq data was considered (Etebari et al., 2015). In addition, splice events were setting for: minimum length of ORF = 100; ignore duplicate or nonspecific read matches; minimum spliced and unspliced reads = 10 and minimum spliced and unspliced coverage ratio = 0.05 were allowed; chimeric or unknown splice signatures were ignored, gene merging distance = 50 bp; minimum reads in gene = 10; and minimum predicted gene length = 200 (Muniz et al., 2021). Then, a single assembly (GTF file) was generated for each trait.

#### 2.2.2 Long noncoding RNA (lncRNA) identification and reads mapping

To identify potential long noncoding RNAs from average of 887,304 predicted transcript (gtf file) models for each trait, Feelnc filter pipeline was used (Wucher et al., 2017). The transcripts with a length shorter than 200 bp, biotype‐coding protein, single‐exon transcripts, and biexonic transcripts having one exon size shorter than 25 bp were discarded (Etebari et al., 2015; Gupta et al., 2019). After filtering, the Feelnc coding potential tool, setting up with default parameters, was used to compute the coding potential score of the model transcripts, classifying them into putative lncRNAs and protein‐coding RNAs. Then, the lncRNA transcript file, containing 5903 transcripts was merged with the bovine genome reference (ARS.UCD1.2; ftp.ensembl.org/pub/release‐100/fasta/bos_taurus/), which was used to create gene and RNA track making.

The RNA‐Sequencing analysis tool (CLC Genomics Workbench environment) was used to align the sequences of each sample against the predicted gene and transcript tracks, using the bovine reference genome ARS.UCD1.2 (ftp://ftp.ensembl.org/pub/release‐100) as map, see more information about assembly parameters in Muniz et al. (2021).

#### 2.2.3 Differential lncRNA expression

Differential expression analyses were performed using the CLC Genomics Workbench software 12.0 (CLC Bio). A two‐group experiments [tender vs. tough tenderness and high vs. low marbling groups] were carried out from each trait. Empirical analysis of DGE tool implemented in CLC workbench was performed for each set up experiment, using the original count values and two parameters related to the estimation of the dispersion were specified: (1) “total count filter cut‐off” >5, that specifies which features should be considered when estimating the common dispersion component; (2) “Estimate tag‐wise dispersions,” which allows a weighted combination of the tag‐wise and common dispersion for each transcript. The Empirical analysis of DGE tool implements the “Exact Test” for two‐group comparisons developed by Robinson and Smyth (2008), which is designed to deal with situations in which many features are studied simultaneously, and where a few biological replicates are available for each of the experimental groups studied. This test also accounts for overdispersion caused by biological variability; more details can be found in CLC manuals (CLC Manuals—clcsupport.com (qiagenbioinformatics.com). Then, transformed expression values were calculated by logarithm transformation (log10), and normalized (Reads Per Kilobase Million (RPKM) normalization method: by Total = 1,000,000) (Muniz et al., 2021). Additionally, the fold change (FC) absolute value >2 and *p*‐value (<0.01) were used, aiming to having the most significant transcripts and filtering from them the lncRNAs.

### 2.3 Enrichment analysis and QLT annotation

The FEElnc classifier tool (FEElnc software) was used to classify lncRNAs as following: type of interactions: Genic, when the lncRNA gene overlaps an RNA gene from the reference annotation file, and intergenic(lincRNA) otherwise; then it is classified into subtypes and locations, which are defined according to orientation of the interactions and localization of the transcription of proximal RNA transcripts, more details can be found in the workflow (Figure S1) and in the FEElnc github database (https://github.com/tderrien/FEELnc). This classification was used to explore the DE lncRNA results separately by category. The GO enrichment analysis tool in Gene Ontology (http://geneontology.org/; release 2020‐10‐09) was performed to identify significant enriched biological annotations and pathways associated with genes related to DE lncRNA, through PANTHER Overrepresentation Test with a cut‐off of *p*‐value < 0.05.

Genomic Annotation in Livestock for positional candidate Loci (GALLO) is an R package developed for the accurate annotation of genes and quantitative trait loci (QTLs) located in regions identified in common genomic analyses performed in livestock (Fonseca, Suárez‐Vega, et al., 2020). Thus, we used the “find_genes_qtls_around_markers” function in the GALLO package implemented in R (https://CRAN.R‐project.org/package=GALLO; Fonseca, Suárez‐Vega, et al., 2020) to annotate QTL overlapping with DE lncRNAs coding regions according to the bovine QTL annotation database. For that, the position of the DE lncRNAs and the bovine QTL annotation database (https://www.animalgenome.org/cgi‐bin/QTLdb/index) were used as inputs. Furthermore, the “find genes qtls around markers” tool is used to annotate of genes and QTLs around candidate regions, its output is a data frame composed of the columns present in the input file and the genes or QTLs mapped within or around (if interval provided) the candidate regions. In our study, we annotated QTLs only within DE lncRNAs coding regions.

## 3 RESULTS

### 3.1 Differentially expressed long noncoding RNA (lncRNA) annotated in the genome reference (ARS.UCD 1.2)

For tenderness and marbling traits, three DE long noncoding RNA were identified (Table 1) respectively, out of these lncRNAs [*ENSBTAT00000071552* and *ENSBTAT00000083689*] were upregulated in tough beef animals in relation to tender beef animals, while the *ENSBTAT00000082179* was downregulated to this same contrast. For marbling, the lncRNA [*ENSBTAT00000068862]* was upregulated, and the lncRNAs [*ENSBTAT00000068862* and *ENSBTAT00000083723*] were downregulated in high marbling animals in relation to low marbling. All these lncRNAs are annotated as novel transcripts in the reference genome bovine (ARS.UCD 1.2), and information about functionality and actions of these lncRNAs are scarce in the literature.

**TABLE 1:** Differentially expressed long noncoding RNA in *Longissimus thoracis* muscle of Nellore cattle divergent to tenderness and marbling traits

Feature ID	Position	Length (bp)	*p*‐Value	FC (log2)^a^
Tenderness				
ENSBTAT00000071552	7:71271560–71273301	1432	4.59^E‐03^	15.63
ENSBTAT00000083689	23:28965394–28967670	1894	1.53^E‐03^	11.16
ENSBTAT00000082179	13:42524409–42525905	927	6.11^E‐04^	−17.89
Marbling				
ENSBTAT00000068862	28:42182000–42191908	2100	3.00^E‐03^	31.13
ENSBTAT00000083723	13:69373088–69374544	1000	2.07^E‐03^	−3.52
ENSBTAT00000069701	25:34335946–34338041	1770	8.27^E‐03^	−6.88

^a^ FC (log2) = Fold change (log2).

### 3.2 Novel differentially expressed long genic noncoding RNA (lncRNA)

For tenderness, seven long genic noncoding RNA were identified as DE in tough beef animals in relation to tender beef animals (Table 2). Three upregulated lncRNA (*lncRNA_595*.*1*; *lncRNA_71*.*3*; and *lncRNA_3440*.*2*), and four downregulated (*lncRNA_3097*.*2*; *lncRNA_775*.*7*; *lncRNA_688*.*5* and *lncRNA_3129*.*5*), in tough beef animals in relation to tender beef animals, were identified. These lncRNAs were located in antisense direction of intronic and exonic regions of potential coding protein transcripts. The three upregulated DE lncRNAs were interacting with mRNAs associated with glutamate receptor subtypes, monoamine plasma membrane transporters, nonvoltage‐gated sodium channels, cardiomyocyte proliferation, as well as ATP‐binding cassette (ABC) transporters, respectively.

**TABLE 2:** Novel long genic noncoding RNA differentially expressed in *Longissimus thoracis* muscle of Nellore cattle divergent for tenderness and marbling traits

Feature ID	Position	Length (bp)	*p*‐Value	FC (log2)^a^	mRNA interaction^b^	Interaction location^c^
Tenderness						
lncRNA_595.1	5:109837504–109845333	1824	9.35^E‐04^	76.53	PICK1	Exonic
lncRNA_71.3	1:124888385–124908431	1202	3.56^E‐03^	73.33	DIPK2A	
lncRNA_3440.2	25:14414100–14420759	2948	2.00^E‐04^	2.57	ABCC1	Intronic
lncRNA_3097.2	22:5307752–5323122	870	1.64^E‐03^	−10.92	GADL1	
lncRNA_775.7	7:18395259–18415135	705	4.00^E‐03^	−13.02	RANBP3	Exonic
lncRNA_688.5	6:117647176–117649672	1359	2.82^E‐03^	−65.93	SLC49A3	
lncRNA_3129.5	22:37436799–37438497	1444	3.60^E‐05^	−159.30	PSMD6	Intronic
Marbling						
lncRNA_2890.1	22:16595982–16621228	5481	1.82^E‐05^	159.05	ENSBTAT00000068041.1	Exonic
lncRNA_2887.7	22:14844549–14861932	3217	8.95^E‐03^	45.93	CCDC13	
lncRNA_646.1	7:3939581–3939994	327	8.13^E‐03^	9.41	MAU2	
lncRNA_512.1	5:84754747–84755610	644	2.79^E‐03^	7.58	KRAS	Intronic
lncRNA_3721.1	4:98918793–98924088	268	4.20^E‐03^	5.68	ENSBTAT00000085778.1	
lncRNA_41.5	1:79994089–80006895	7982	9.23^E‐03^	−2.21	MASP1	
lncRNA_41.4	1:79994089–80006895	8997	6.49^E‐03^	−2.93	MASP1	
lncRNA_3191.1	25:26694902–26695467	369	1.06^E‐03^	−17.67	ITGAL	Exonic

^a^ FC (log2) = Fold change (log2).

^b^ mRNA interaction = lncRNA gene overlaps an mRNA gene from the bovine reference annotation (ARS.UCD1.2).

^c^ Interaction location = are defined according to the type of interactions (genic or intergenic), assuming that genic lncRNA are overlapping a RNA transcript (mRNA/gene), then it can be further classified in: exonic (overlaps exon regions) or intronic (overlaps intron regions).

For marbling, eight genic lncRNA were differentially expressed in high marbling group in relation low marbling group (Table 2). These lncRNAs were located in antisense direction of intronic and exonic regions of their gene interactions. Out of those, five lncRNAs [*lncRNA_2890*.*1*, *lncRNA_2887*.*7*, *lncRNA_646*.*1*, *lncRNA_512*.*1*, and *lncRNA_3721*.*1*] were upregulated in high marbling beef compared to low marbling beef animals. These novel genic lncRNA were associated with potential coding protein related to Melorheostosis, a rare skeletal abnormality that causes growth of new bone tissue on top of existing. The downregulated novel genic lncRNAs [*lncRNA_41*.*5*, *lncRNA_41*.*4* and *lncRNA*_*3191*.*1*] in high marbling in relation to low marbling beef animals (Table 2) were associated with the *MASP1* (Mannan Binding Lectin Serine Peptidase 1) and *ITGAL* (Integrin Subunit Alpha L). The *MASP1* encodes a serine protease that functions as a component of the lectin pathway of complement activation, and the *ITGAL* encodes an integrin, that are heterodimeric integral membrane proteins composed of an alpha chain and a beta chain.

### 3.3 Novel differentially expressed long intergenic noncoding RNA

For tenderness, 19 novel long intergenic noncoding RNA (lincRNA) were differentially expressed in tough beef animals in relation to tender beef animals (Table 3), out of them, nine were upregulated and 10 downregulated, respectively. Most of these lincRNAs were located in antisense direction (81.25%) in relation to their gene interactions. The upregulated novel lincRNA (Table 3) identified as DE for tenderness trait in tough beef animals in relation to tender were associated with the coding proteins related to RNA binding protein, metalloprotease, ubiquitin‐protein ligase, ribosomal protein, and phospholipase. In addition, downregulated novel lincRNAs (Table 3) were associated with the genes related to protein classes, such as transcription co‐factor, dehydrogenase homeodomain transcription factor, DNA‐ directed DNA polymerase, and oxidase.

**TABLE 3:** Novel long intergenic noncoding RNA differentially expressed in *Longissimus thoracis* muscle of Nellore cattle divergent for tenderness trait

lncRNA located in antisense direction^a^							
Feature ID	Position	Length (bp)	*p*‐Value	FC (log2)^b^	mRNA interaction^c^	Distance^d^	Interaction location^e^
lncRNA_3811.1	X:134859201–134903897	2289	1.09^E‐05^	276.44	PNPLA4	91	Upstream
lncRNA_1173.1	10:91746240–91758777	555	8.69^E‐03^	140.79	ENSBTAT00000054915.3	2350	Downstream
lncRNA_654.1	6:58750827–58774420	1447	2.07^E‐03^	80.55	UBE2K	153	Upstream
lncRNA_2375.1	16:73105964–73107141	819	7.64^E‐03^	41.86	SERTAD4	283	
lncRNA_5303.3	27:6111664–6117311	523	3.30^E‐03^	23.78	ZNF705A	2672	
lncRNA_226.1	2:131319247–131324359	3423	1.47^E‐05^	10.16	ECE1	249	
lncRNA_395.6	3:120405707–120410738	2447	2.37^E‐03^	8.93	HDLBP	13157	Downstream
lncRNA_2328.3	16:43978508–43985213	3149	4.79^E‐03^	4.68	SLC25A33	31809	Upstream
lncRNA_2840.5	19:35588271–35591518	695	7.08^E‐03^	2.27	NME1	2655	Downstream
lncRNA_903.2	8:57429997–57440234	1070	9.42^E‐03^	−3.37	TLE1	93665	
lncRNA_3591.5	27:15765603–15783342	2530	7.61^E‐04^	−3.57	SORBS2	7056	
lncRNA_3797.2	X:119425885–119431843	2282	2.01^E‐03^	−6.75	SAT1	77	
lncRNA_36.3	1:72000667–72002124	658	4.85^E‐04^	−9.41	BDH1	131	Upstream
lncRNA_36.1	1:72000667–72002124	673	5.62^E‐04^	−9.63			
lncRNA_2518.1	18:23221114–23229258	2180	1.35^E‐03^	−10.57	IRX5	5503	
lncRNA_975.6	9:38910915–38912274	575	6.25^E‐03^	−34.16	REV3L	18167	
lncRNA_624.1	5:119828031–119838020	584	2.88^E‐06^	−180.93	SCO2	3782	

lncRNA located in sense direction^a^							
Feature ID	Position	Length(bp)	*p*‐Value	FC (log2)^b^	mRNA interaction^c^	Distance^d^	Location^e^
lncRNA_3291.4	23:27782209–27785626	2247	1.77^E‐03^	−6.28	ENSBTAT00000055663.3	10569	Upstream
lncRNA_3586.2	27:6116986–6117643	396	7.84^E‐03^	−27.44	ZNF705A	2340	

^a^ Direction of transcription of proximal RNA transcripts.

^b^ FC (log2) = Fold change (log2).

^c^ mRNA interaction = the closest mRNA to the lincRNA.

^d^ Distance = distance between lincRNA and closest mRNA in the bovine reference annotation (ARS.UCD1.2).

^e^ Interaction location = are defined according to the type of interactions (genic or intergenic), assuming that intergenic lncRNA are not overlapping any RNA transcript (mRNA/gene), then it can be further classified in: upstream (the lncRNA is upstream transcribed in head‐to‐head or tail‐to‐tail orientation with RNA partner or yet both same orientation) or downstream (the lncRNA is downstream transcribed in head‐to‐head or tail‐to‐tail orientation with RNA partner or yet both in same orientation).

Regarding the marbling trait, 37 novels differentially expressed lincRNAs were found (Table 4). From these, 21 were upregulated and 16 downregulated in high marbling beef animals in relation to low marbling beef animals, respectively. These RNA‐like intergenic transcripts (lincRNAs) were located mostly in antisense position of protein‐coding (70.27%) transcripts. The novel DE lincRNAs upregulated in high marbling animals compared to low marbling were closely related to genes associated with C2H2 zinc finger transcription factor and C4 zinc finger nuclear receptor, dehydrogenase, lipase, transcription factor binding and translation initiation factor, calcium ion binding, microtubule, actin and Rab GTPase bindings, oxidoreductase that promotes fatty‐acid oxidation, and mitochondrial carrier proteins. Additionally, the downregulated transcripts (Table 4) in high compared to low marbling beef animals were associated with genes related to degradation of post‐translationally modified proteins in lysosomes, mTOR signaling, and malonyl‐CoA decarboxylase activity, and actin binding, and calmodulin binding.

**TABLE 4:** Novel long intergenic noncoding RNA differentially expressed in *Longissimus thoracis* muscle of Nellore cattle divergent for marbling trait

lncRNA located in antisense direction^a^							
Feature ID	Position	Length (bp)	*p*‐Value	FC (log2)^b^	mRNA interaction^c^	Distance^d^	Interaction location^e^
lncRNA_3465.1	X:52825495–52854491	3487	3.50^E‐08^	356.50	FAM199X	14733	Upstream
lncRNA_1000.1	10:36713922–36719606	3627	4.56^E‐06^	341.29	CHP1	36	
lncRNA_535.1	5:109301621–109305580	3039	7.31^E‐04^	142.83	MICAL3	27014	
lncRNA_88.3	1:148598117–148604626	5496	7.61^E‐05^	129.69	MORC3	274	
lncRNA_918.1	9:88227847–88229124	1123	2.87^E‐04^	126.60	AKAP12	61489	
lncRNA_10.1	1:43009042–43024251	485	8.29^E‐05^	81.06	ST3GAL6	23725	
lncRNA_828.6	8:66935095–66944775	3964	2.83^E‐03^	72.59	LPL	44890	
lncRNA_2784.1	20:35573793–35575950	1522	3.20^E‐04^	67.26	OSMR	2283	
lncRNA_2672.1	19:38565495–38578920	1062	1.32^E‐03^	44.53	SP2	382	
lncRNA_1932.1	13:19967060–19971062	3886	7.04^E‐03^	13.00	ITGB1	560	
lncRNA_3530.1	1:72000693–72002124	689	7.34^E‐03^	7.78	BDH1	157	
lncRNA_4400.1	16:43978462–43985298	3280	5.17^E‐04^	6.11	SLC25A33	31763	
lncRNA_2303.3	17:67362408–67365283	943	8.07^E‐03^	−8.51	ENSBTAT00000072011.1	9299	
lncRNA_889.2	9:38910924–38930012	1778	1.62^E‐03^	−53.93	REV3L	429	
lncRNA_586.7	6:24479496–24525896	3488	3.05^E‐03^	−75.45	DDIT4L	545	
lncRNA_2350.5	18:10228225–10234398	3735	1.37^E‐03^	−84.76	MLYCD	5926	
lncRNA_256.7	3:16734412–16737753	2805	8.73^E‐04^	−89.42	SNAPIN	132	
lncRNA_1053.5	10:88238796–88243059	3903	1.08^E‐03^	−92.36	IRF2BPL	135	
lncRNA_970.1	10:21488702–21490158	282	1.07^E‐03^	−101.95	MYH7	252	
lncRNA_889.1	9:38910924–38930012	994	6.68^E‐04^	−119.13	REV3L	440	
lncRNA_589.1	6:25791706–25795076	1659	1.03^E‐09^	291.23	EIF4E	85959	Downstream
lncRNA_4219.1	12:14130644–14136514	859	7.34^E‐03^	31.03	LACC1	26800	
lncRNA_2228.1	16:75493995–75508021	1314	4.62^E‐03^	18.64	CD34	4737	
lncRNA_3212.4	25:34409653–34414866	813	7.95^E‐04^	−7.03	SSC4D	1290	
lncRNA_724.2	7:21501677–21502701	505	9.80^E‐03^	−29.76	MKNK2	214	
lncRNA_3383.2	29:37152043–37153348	1196	1.45^E‐03^	−62.21	MS4A10	4963	

lncRNA located in sense direction^a^							
Feature ID	Position	Length (bp)	*p*‐Value	FC (log2)^b^	mRNA interaction^c^	Distance^d^	Location^e^
lncRNA_556.1	5:116431233–116437621	2242	6.64^E‐04^	111.76	PPARA	1366	Upstream
lncRNA_680.1	7:14216103–14217002	577	2.67^E‐03^	98.55	ZNF177	15982	
lncRNA_718.1	7:20813940–20824430	269	5.73^E‐04^	54.36	ENSBTAT00000017099.6	2153	
lncRNA_845.1	8:88589631–88592276	2611	8.96^E‐03^	48.49	SEMA4D	34892	
lncRNA_909.1	9:87117711–87120792	1913	5.07^E‐03^	38.35	ENSBTAT00000053728.3	616	
lncRNA_4017.2	9:88235260–88245618	3880	7.09^E‐03^	8.99	AKAP12	44995	
lncRNA_3124.2	25:564383–564914	355	9.99^E‐03^	−15.65	ENSBTAT00000072927.1	6002	
lncRNA_951.1	10:15078518–15093449	239	3.89^E‐03^	−30.14	CLN6	10831	
lncRNA_3150.1	25:2579931–2582254	1668	1.85^E‐03^	−42.73	ENSBTAT00000075295.1	632	
lncRNA_727.4	7:26317030–26324342	6737	1.08^E‐03^	−113.23	CTXN3	3181	
lncRNA_844.4	8:86704258–86720514	9628	1.81^E‐04^	−2.97	AUH	18099	Downstream

^a^ Direction of transcription of proximal RNA transcripts.

^b^ FC (log2) = Fold change (log2).

^c^ mRNA interaction = the closest mRNA to the lincRNA.

^d^ Distance = distance between lincRNA and closest mRNA in the bovine reference annotation (ARS.UCD1.2).

^e^ Interaction location = are defined according to the type of interactions (genic or intergenic), assuming that intergenic lncRNA are not overlapping any RNA transcript (mRNA/gene), then it can be further classified in: upstream (the lncRNA is upstream transcribed in head‐to‐head or tail‐to‐tail orientation with RNA partner or yet both same orientation) or downstream (the lncRNA is downstream transcribed in head‐to‐head or tail‐to‐tail orientation with RNA partner or yet both in the same orientation).

### 3.4 Enrichment analysis and QTL annotation

Functional classification of GO terms (i.e., molecular function, biological process, and cellular component) was performed for genes associated with DE lncRNAs for tenderness (Figure S2) and marbling (Figure S3) traits.

**FIGURE 1:**
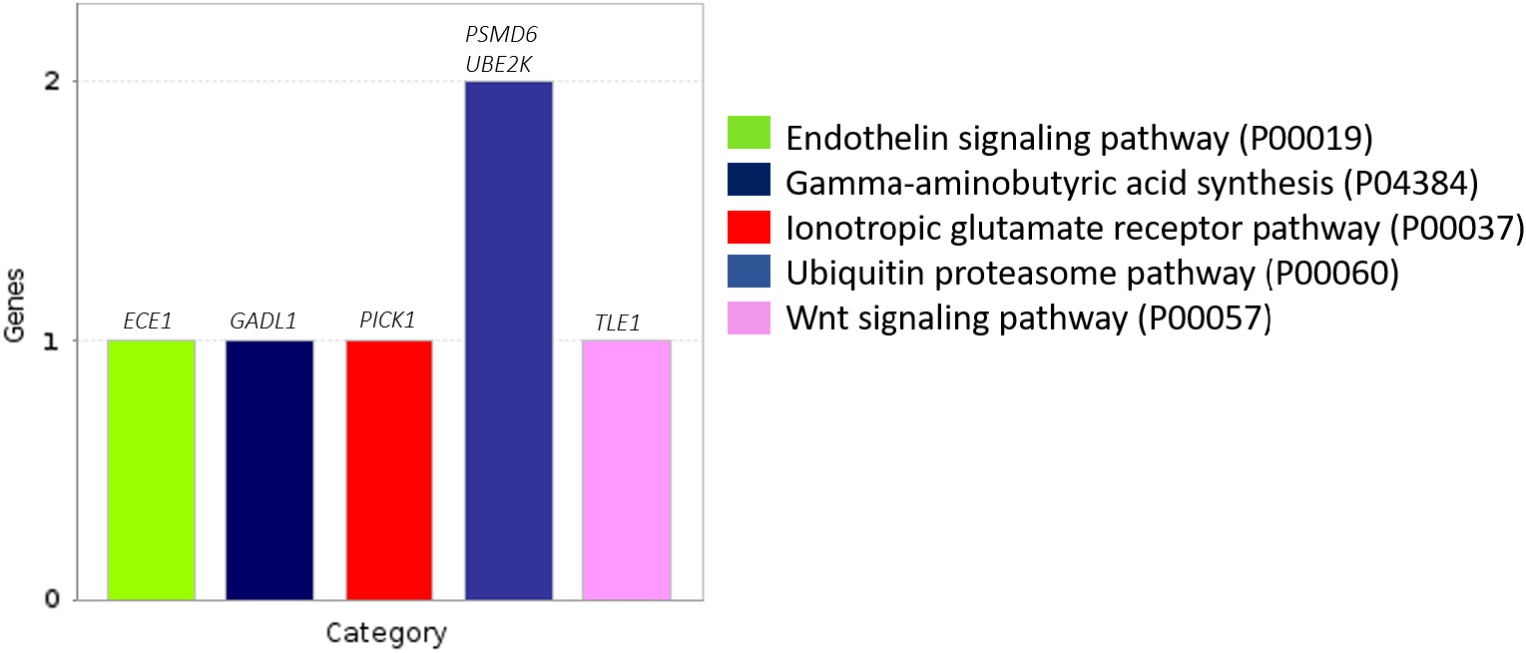
Panther pathways associated (*p*‐value < 0.05) with tenderness in beef cattle

For tenderness, five significant pathways (*p*‐value < 0.05) were identified (Figure 1), which were enriched by six genes (*ECE1*, *GADL1*, *PICK1*, *PSMD6*, *UBE2K*, and *TLE1*) associated with the DE lncRNAs identified in this study.

**FIGURE 2:**
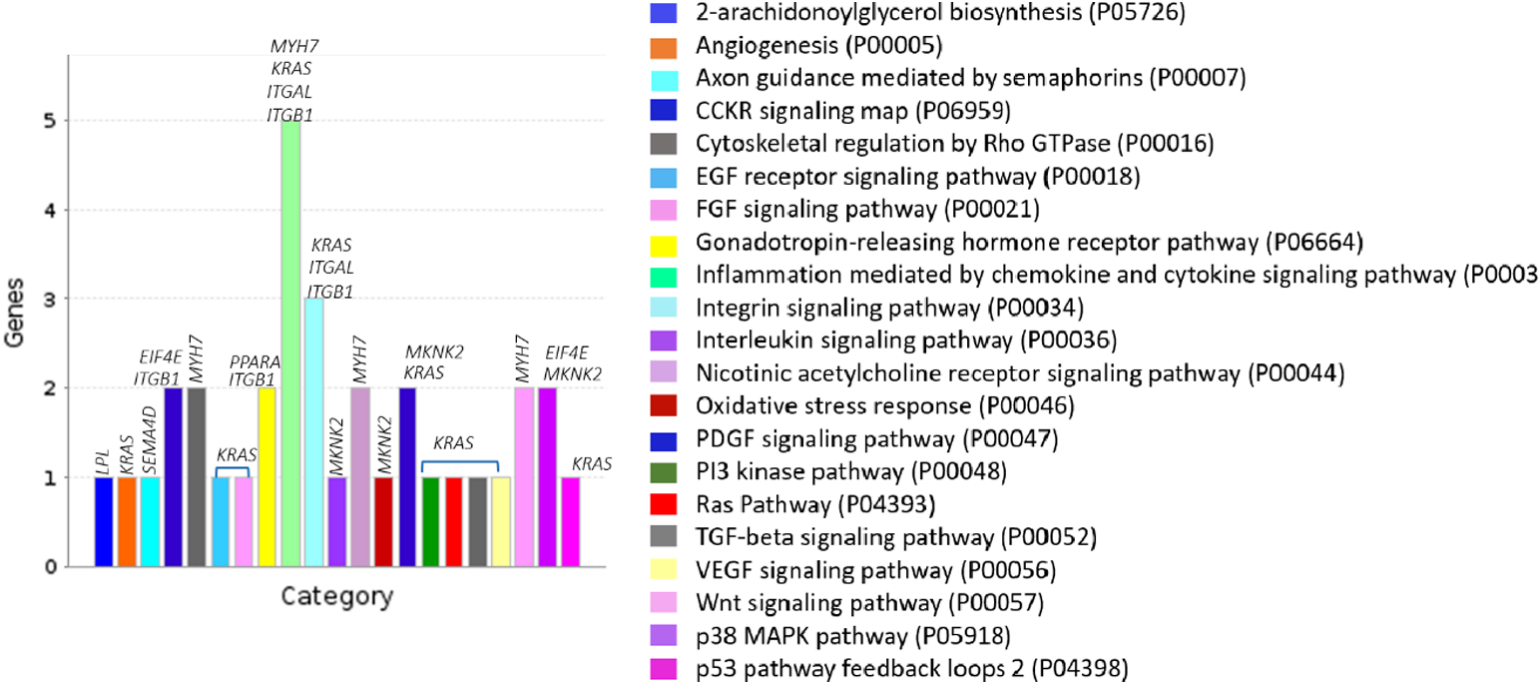
Panther pathways associated (*p*‐value < 0.05) with marbling in beef cattle

For marbling, 21 significant pathways (*p*‐value < 0.05) were identified (Figure 2), which were enriched by nine genes (*LPL*, *KRAS*, *SEMA4D*, *ITGB1*, *EIF4E*, *MYH7*, *PPARA*, *ITGAL*, and *MKNK2*) associated with the DE lncRNAs identified in this study.

**FIGURE 3:**
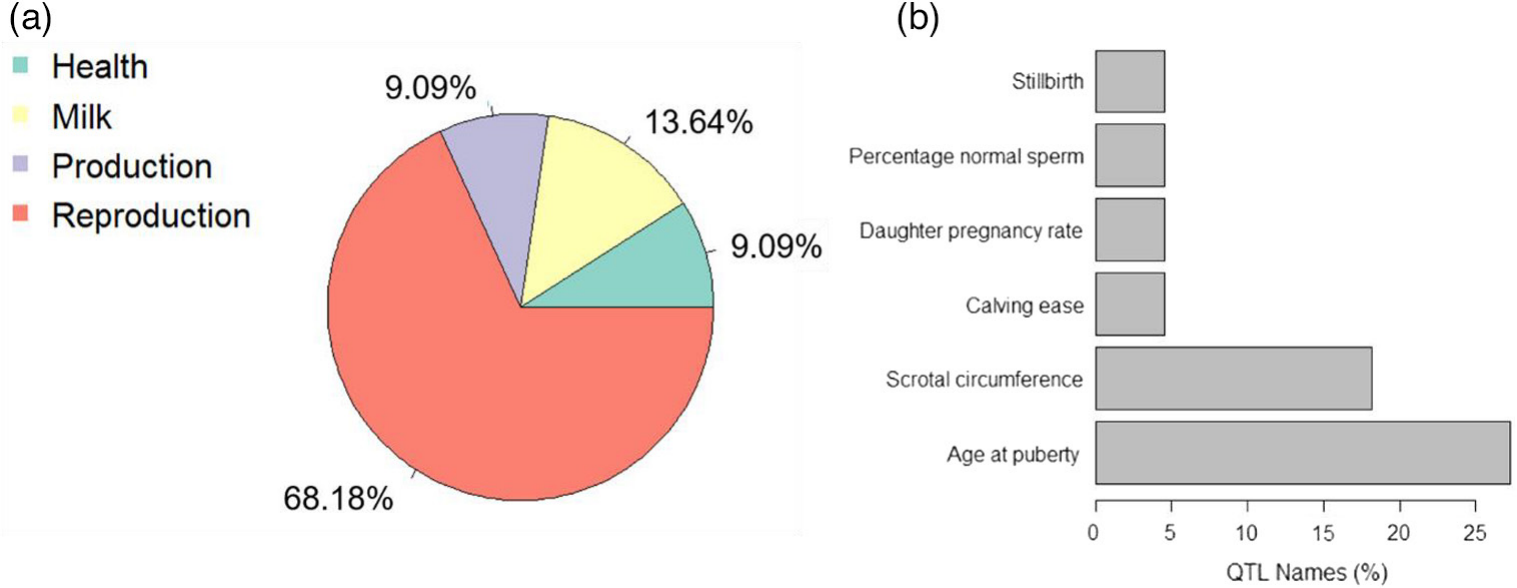
QTL annotation in transcription regions associated with marbling and tenderness beef traits. (a) Total QTLs annotated for DE lncRNA regions; (b) QTLs annotated for reproductive traits

Quantitative trait loci annotation analysis was performed for genome regions codifying DE lncRNA between divergent phenotypes of tenderness and marbling beef traits. Interestingly, 68.18% of QTLs in overlap with the DE lncRNA regions, are related to reproductive traits (Figure 3a). Furthermore, age at puberty (>25% of QTLs) and scrotal circumference (~18% of QTL) (Figure 3b) were the most representative traits within total number of QTLs annotated for reproductive traits. The QTLs annotated for lncRNA regions identified for tenderness were located on the chromosomes 14 and 25, and for marbling on the chromosomes 5, 8, 25, and X.

## 4 DISCUSSION

Some DE lncRNAs identified in this study have been annotated in the bovine genome reference (ARS.UCD1.2) (Table 1). All these lncRNAs were annotated as novel transcripts at the bovine genome reference, thus no information has been found about interactions and functionality of them. However, due to their significantly different expression levels (Table 1) in animals divergent for tenderness and marbling traits, we could hypothesize that these lncRNAs are important regulators of coding‐protein genes, contributing to the expression of these phenotypes.

The vast majority of the genome produces numerous lncRNAs, which comprise various RNA species longer than 200 nucleotides (nt) that are not translated into proteins (Yao et al., 2019). Studies indicate that lncRNA structure is one of the most critical factors determining its interactions and thus its specific function (Duran et al., 2019; Gupta et al., 2019). Usually, lncRNAs are categorized according to their genomic locations in relation to the nearest protein‐coding. Here, we categorized lncRNAs according to their genic location and identified two general categories: the genic lncRNA, for tenderness and for marbling traits (Table 2), and intergenic lncRNAs, for tenderness (Table 3) and for marbling (Table 4) traits.

Genic lncRNAs are long noncoding RNAs overlapping coding‐proteins, intersecting gene exons, introns, or an entire gene respectively, and affect the functionality of their associated mRNA (Duran et al., 2019), being further classified in exonic and intronic. For tenderness, we observed four exonic lncRNA (*lncRNA_595*.*1*, *lncRNA_71*.*3*, *lncRNA_775*.*7* and *lncRNA_688*.*5*) overlapping part of the genes such as: *PICK1*, *DIPK2A*, *RANBP3*, and *SLC49A3*; and three intronic lncRNAs (*lncRNA_3440*.*2*, *lncRNA_3097*.*2*, and *lncRNA_3129*.*5*) intersecting the genes: *ABCC1*, *GADL1*, and *PSMD6* (Table 2). All these lncRNAs were located in antisense direction of their associated mRNA/genes. Some of the genes associated with our DE lncRNA, such as genes *PICK1*, *GADL1*, and *PMD6* were enriched in the pathways associated with Ionotropic glutamate receptor, gamma‐aminobutyric acid synthesis and ubiquitin proteasome, respectively (Figure 1). The *PCK1* is a main control point for the gluconeogenesis regulation and was found involved in Inotropic glutamate receptor pathways (Figure 1), which are receptors ligand‐gated ion channels permissive to cation flux across the cell membrane (Kim et al., 2020). A study comparing muscle and intramuscular fat tissue transcriptome analysis (Huang et al., 2020) reveals that *PCK1* gene was upregulated in adipose tissue compared to muscle tissue in both buffalo and cattle. However, it seems to be more abundant in muscle tissue than in adipose tissue in cattle and may be a potential gene affecting IMF deposition, and meat quality traits. In this study, the lncRNA, *lncRNA_595*.*1*, associated with *PCK1*, was highly upregulated in tough meat animals in relation to tender, which may be affecting the *PCK1* expression levels, contributing to the regulation of the complex transcriptional network behind the diversity of meat quality phenotype expression profiles.

The lncRNA (*lncRNA_3097*.*2*) was downregulated in tough beef animals compared to tender (Table 2) and was associated with a *GADL1* gene. Some studies have shown that *GADL1* overexpression suppressed cell migration, and are associated with morphological changes in these cells (Wu, Chen, Lee, et al., 2019; Wu, Chen, Liu, et al., 2019). This gene encodes a glutamate decarboxylase like 1 protein, which is part of a large family of protein responsible for catalyzing the production of gamma‐aminobutyric acid from l‐glutamic acid. This gene was enriched to one of the pathways (gamma‐aminobutyric acid synthesis) associated with meat tenderness (Figure 1). Gamma‐Aminobutyric acid (GABA) synthesis (l‐glutamic acid +H+ 4 GABA +COz) is rapidly stimulated by a variety of stress conditions (Crawford et al., 1994) (e.g., cell stress originated by oxygen lacking) to enhance cell's resistance. Chun et al. (2014), studying the effects of γ‐aminobutylic acid (GABA) on the quality and sensory properties of meat products, revealed that a GABA percentual increase contributed to a greater tenderness index, and can be used to replace NaCl of a meat product. In beef cattle, Mashima et al. (2019) correlated muscle fiber type and free amino acids (e.g., glutamine) in Japanese Black steers, expecting to find more free amino acids in slow‐twitch fibers than in fast‐twitch fibers due to the slow‐twitch fiber mitochondria content. They reported a correlation of 0.11% and 0.63% between the proportions of myosin (MyHC1), a main muscle protein and highly expressed in slow‐twitch (type 1) muscle fibers, and glutamine and gamma‐aminobutyric acid, free amino acid contents in muscle, suggesting that an increase in slow‐twitch fiber content induces an increase in the total free amino acid content, possibly due to high oxidative metabolism of bovine muscles. The intronic DE lncRNA (*lncRNA_3129*.*5*) was downregulated in tough beef animals compared to tender and associated with the *PMD6* gene (Table 2), that enriched to ubiquitin–proteasome pathway (Figure 1). While ubiquitin–proteasome system genes can affect muscle mass including myofibrillar protein degradation, and myogenesis inhibition (Khalil, 2018). Regardless, there is still few information about how the lncRNAs identified in this study, have been acting together with genes, and how they may be contributing to meat tenderness. Therefore, future studies need to be performed to establish the function of lncRNAs in muscle tissue of tender beef animals.

For marbling, we observed four exonic lncRNA (*lncRNA_2890*.*1*, *lncRNA_2887*.*7*, *lncRNA_646*.*1*, and *lncRNA_3191*.*1*) that were overlapping part of genes such as, *CCDC13*, *MAU2*, and *ITGAL*, and four intronic lncRNAs (*lncRNA_512*.*1*, *lncRNA_3721*.*1*, *lncRNA_41*.*5*, and *lncRNA_41*.*4*) intersecting part of genes (e.g., *KRAS* and *MASP1*) (Table 2). These genes (*KRAS* and *ITGAL*) are enriched in the pathways associated with integrin signaling, EGF receptor, FGF and PDGF signaling, Pi3 kinase, Ras pathway, TGF‐beta and VEGF signaling, and inflammation mediated by chemokine and cytokine, respectively (Figure 2). The *KRAS* (KRAS Proto‐Oncogene, GTPase) encodes a protein that is a member of the small GTPase superfamily. The small GTPase, p21ras, is a known target of reactive nitrogen and oxygen species (RNS/ROS) and may be regulated by oxidative post‐translational modification of cysteines (Clavreul et al., 2006). This gene was associated with the intronic lncRNA (*lncRNA_512*.*10*), upregulated in high marbling animals compared with low marbling animals. This association may play an important role in intramuscular fat deposition, since this DE lncRNA seems to act in several pathways (Figure 2) and may be a key gene, triggering multiple complex biological processes. Zhang et al. (2020) investigated a novel lncRNA, lnc403, involved in bovine skeletal muscle myogenesis by mediating KRAS/Myf6. They reported that this lncRNA could inhibit skeletal muscle satellite cell differentiation and positively regulate the expression of the interacting protein *KRAS* that affects skeletal muscle cell differentiation, suggesting that the lnc403 probably is a key regulatory element in the bovine myoblasts’ differentiation through multi‐pathway network regulation mode. In general, the pathways associated with *KRAS* gene are connected to each other. For example the FGF signaling, generally, follows one of two transduction pathways: RAS/MAP kinase and PI3/AKT, improper activation of those can contribute to unregulated cell growth causing many skeletal abnormalities (Teven et al., 2014).

The exonic lncRNA (*lncRNA_3191*.*1*) associated with the *ITGAL* gene, was downregulated in high marbling beef animals in relation to low (Table 2). The *ITGAL* encodes the integrin alpha L chain. The pathways associated with this gene are involved in the control and direct trafficking and migration of immune cells (e.g., inflammation mediated by chemokine and cytokine signaling). The cytokines are plurifunctional mediators of cellular communication and they can activate specific receptor coupled cellular signal transduction pathways such as the JAK/STAT tyrosine kinase signaling cascade (Campbell, 2005). In addition, integrin signaling pathway, that was enriched by *KRAS* and *ITGAL* genes, is involved in intracellular signals in response to the extracellular matrix including cellular shape, mobility, and mediate the progression through the cell cycle (Harburger & Calderwood, 2009). Thus, these lncRNAs and their mRNA association should be further investigated to better understanding their function and integrative mechanisms for marbling in Nellore cattle.

The lincRNAs are characterized by a lack of physical overlap between lincRNAs and protein‐coding genes. They have various features that distinguish them from mRNA‐encoding genes and they perform functions such as remodeling chromatin and genome architecture, RNA stabilization, and transcription regulation Zhang et al. (2019). For tenderness, nine DE were upregulated lincRNA in tough meat animals compared to tender, while 10 were downregulated. These lincRNAs were related to several genes, among them the *ECE1*, *UBE2K*, and *TLE1* (Table 3). These genes were enriched in the three pathways associated with endothelin, ubiquitin–proteasome, and Wnt signaling (Figure 1). The *ECE1* (Endothelin converting enzyme 1) is involved in proteolytic processing of endothelin precursors for biologically active peptides. It is a key enzyme to generate Endothelin (ET‐1), a potent vasoconstrictor peptide, and has been related to hypoxia resistance in pigs (Wang et al., 2015) and human (Kon et al., 2014). Hypoxia is an important modulator of endurance exercise‐induced oxidative adaptations in skeletal muscle (Kon et al., 2014). The ubiquitin–proteasome has been related to muscle atrophy; thus, a lot of ubiquitin–proteasome system genes are involved in different processes affecting muscle mass, including myofibrillar protein degradation and myogenesis inhibition (Khalil, 2018). In some gene expression studies on meat tenderness in Nellore cattle, genes related to ubiquitin metabolism have been identified (Fonseca et al., 2017; Gonçalves et al., 2018; Muniz et al., 2020, 2021). Wnt signaling is one of the most important developmental signaling pathways, and controls cell fate decisions and tissue patterning during early embryonic and later development (Buechling & Boutros, 2011). The activation of Wnt signal transduction pathways can regulate diverse processes including cell proliferation, migration, polarity, and differentiation (Eisenmann, 2005). In pigs, Zhu et al. (2016) studying RNA‐seq transcriptome of extensor digitorum longus and soleus muscles, have identified 29 enriched genes in the Wnt signal pathway and associated it with myofiber type determination, in which meat tenderness is, probably, included. Therefore, our findings can be important new sights for meat tenderness studies and suggest further investigation of regulatory elements acting into these pathways to better understand the roles of lncRNAs.

For marbling, 26 lincRNA were DE; of those, 15 were upregulated while 11 were downregulated. These lincRNAs were associated with various mRNA related to meat quality, calcium binding, muscle structure, skeletal muscle hypertrophy, lipase, and endothelial cell migration (Cao et al., 2018; Figueiredo, 2019; Pratt et al., 2013). Among those, the lincRNAs, *lncRNA_828*.*6*, *lncRNA_ 845*.*1*, *lncRNA_589*.*1*, *lncRNA_1932*.*1*, *lncRNA_970*.*1*, *lncRNA_556*.*1*, and *lncRNA_724*.*2* were closely associated with the genes, *LPL*, *SEMA4D*, *EIF4E*, *ITGB1*, *MYH7*, *PPARA*, and *MKNK2*, respectively, which were enriched to important pathways, such as 2‐arachidonoylglycerol biosynthesis, axon guidance mediated by semaphorins, cytoskeletal regulation by Rho GTPase, interleukin signaling, oxidative stress response, PDGF and Wnt signaling, p38 MAPK and CCKR signaling map pathways (Figure 2). The most part of these pathways is connected to each other, which triggers various biological processes contributing to multiple molecular functions (Figures S2 and S3) that are involved in processes such as lipid metabolism (Jesudason & Wittert, 2008), neuromuscular junctions restoration during muscle repair (Daneshvar et al., 2020), skeletal muscle regeneration (Bryan et al., 2005), skeletal maturation delaying (Balasubramanian & Crowley, 2019), skeletal muscle hypertrophy (Boppart & Mahmassani, 2019), increased glucose metabolism and reduced inflammation in skeletal muscle (Dagdeviren et al., 2016), and collagen synthesis (Purslow et al., 2012). Therefore, the regulatory function of these lincRNAs into these pathways to expression of marbling and tenderness traits should be furthermore investigated.

The OMICs sciences have contributed greatly to the knowledge of complex traits, such as tenderness and marbling (Braz et al., 2019; Magalhães et al., 2016; Santos et al., 2019), revealing genes and their mechanisms in the genomic and transcriptional levels. However, the integration of results from these multiple approaches is still challenging, magnifying the need for efficient and accurate integrative methods to puzzle out the relationship between transcriptional regulation and several phenotypes, allowing to identify genetic variants associated with changes in the expression of economically important traits. In this study, we have performed QTL annotation into DE lncRNA regions aiming to point out QTL regions that may be selected to further meat quality investigations. As shown in Figure 3, the most part of QTLs annotated are related to reproductive traits (Figure 3a), being age at puberty (>25% of QTLs) and scrotal circumference (~18% of QTL) (Figure 3b), the most representative traits within total number of QTLs annotated for reproductive traits. These results may be suggesting regions carrying QTLs in the chromosomes 5, 8, 14, 25, and X, with possible pleiotropic effect on meat quality and reproductive traits. Genomic and transcriptomics studies performed for meat tenderness and marbling traits, have reported genic regions and expressed genes associated with these traits in Nellore cattle, which are located in the chromosomes 5, 8, 14, 25, and X (Berton et al., 2016; Castro et al., 2017; Fonseca et al., 2017; Magalhães et al., 2016). On the other hand, several studies have shown association among these same regions to reproductive traits. Melo et al. (2018) identified SNP in the chromosomes BTA14 and 25, associated with reproductive traits such as age at first calving and scrotal circumference in beef cattle (Nellore and Brahman). Melo et al. (2017) reported several genes associated with reproductive events involved in metabolism, p53 signaling, Axon guidance, and ubiquitin pathways, some of these pathways had been associated with meat quality traits in this study. Irano et al. (2016) identified 10 genomic windows located on chromosomes 5, 8, and 14 associated with the occurrence of early pregnancy and scrotal circumference. Thus, these results may be indicating a possible genetic correlation between meat quality (e.g., tenderness and marbling) and reproductive traits (e.g., scrotal circumference and age at puberty), however, studies reporting genetic correlations between meat quality and reproductive traits are scarce, which does not allow us to further speculate about those associations and how these QTLs can be affecting both, meat quality and reproductive traits.

## 5 CONCLUSION

This study targeted specific genomic regions of lncRNA codification and pathways that showed a multifactorial interaction that resembles transcription factor recruitment at specific genomic sites. Thus, the findings obtained further advance our understanding of the transcriptional regulation roles of lncRNAs and the growing importance of these molecules in the muscle biology system and for meat quality traits. Therefore, further investigation to understand the interaction between lncRNA and mRNAs affecting meat quality traits, and possible pleiotropic effects regarding reproductive traits are needed.

## CONFLICT OF INTEREST

The authors have no conflicts of interest to declare.

## Supporting information

Fig S1‐3

## Data Availability

The dataset utilized in this study belongs to a Qualitas Nelore breeding program company and could be available on request. The authors do not have the authorization to share the data.
