# Supplementary material for: Characterization of novel lncRNA muscle expression profiles associated with meat quality in beef cattle
Source: Evol Appl. 2022 Mar 25;15(4):706–18. doi: 10.1111/eva.13365 (PMC9046762; doi:10.1111/eva.13365)
Supplement: Supplementary file 1 — Fig S1‐3 [file EVA-15-706-s001.docx]

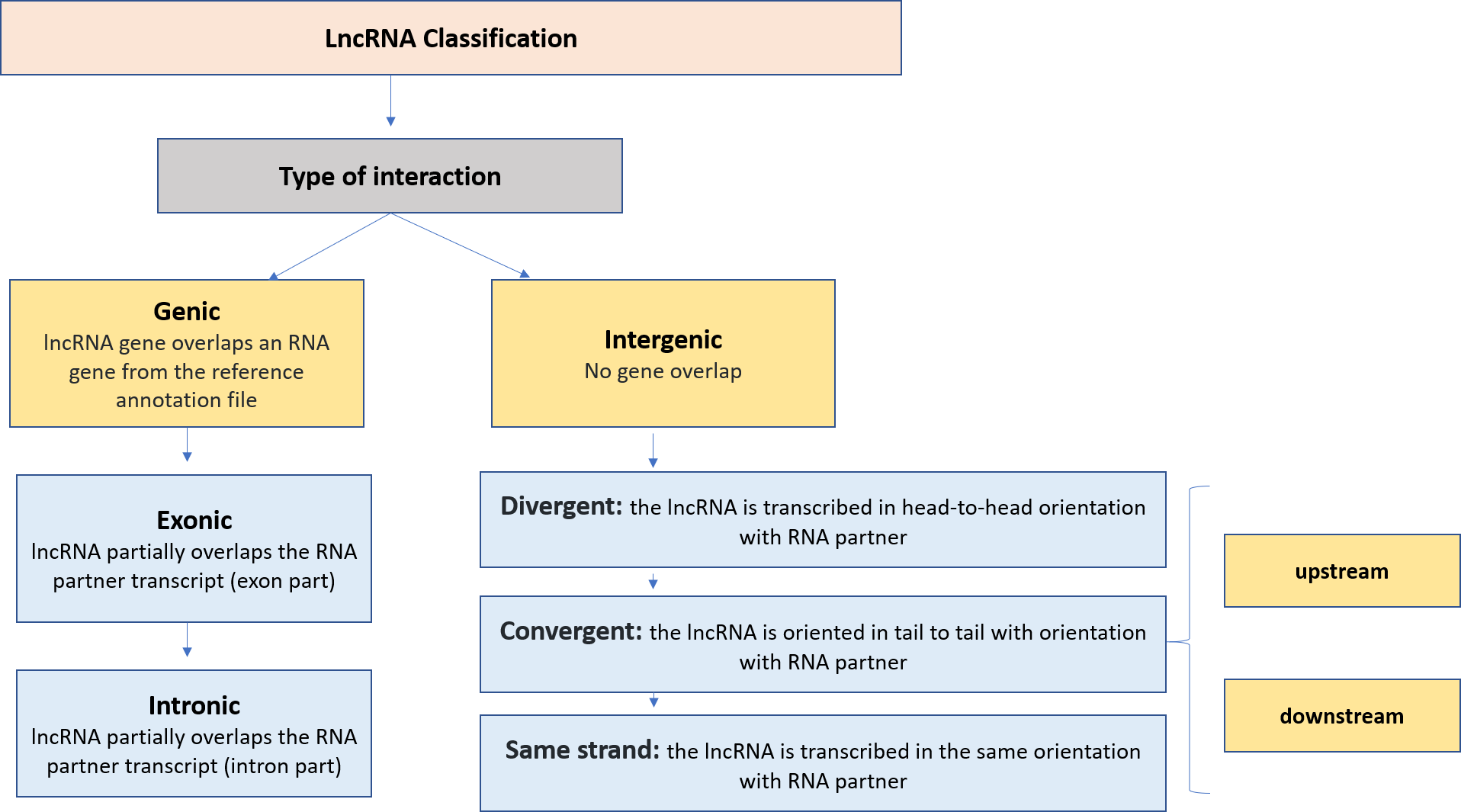


**FIGURE S1.** Workflow of DE lncRNA classification according with their location in reference genome in relation to RNA interactions.


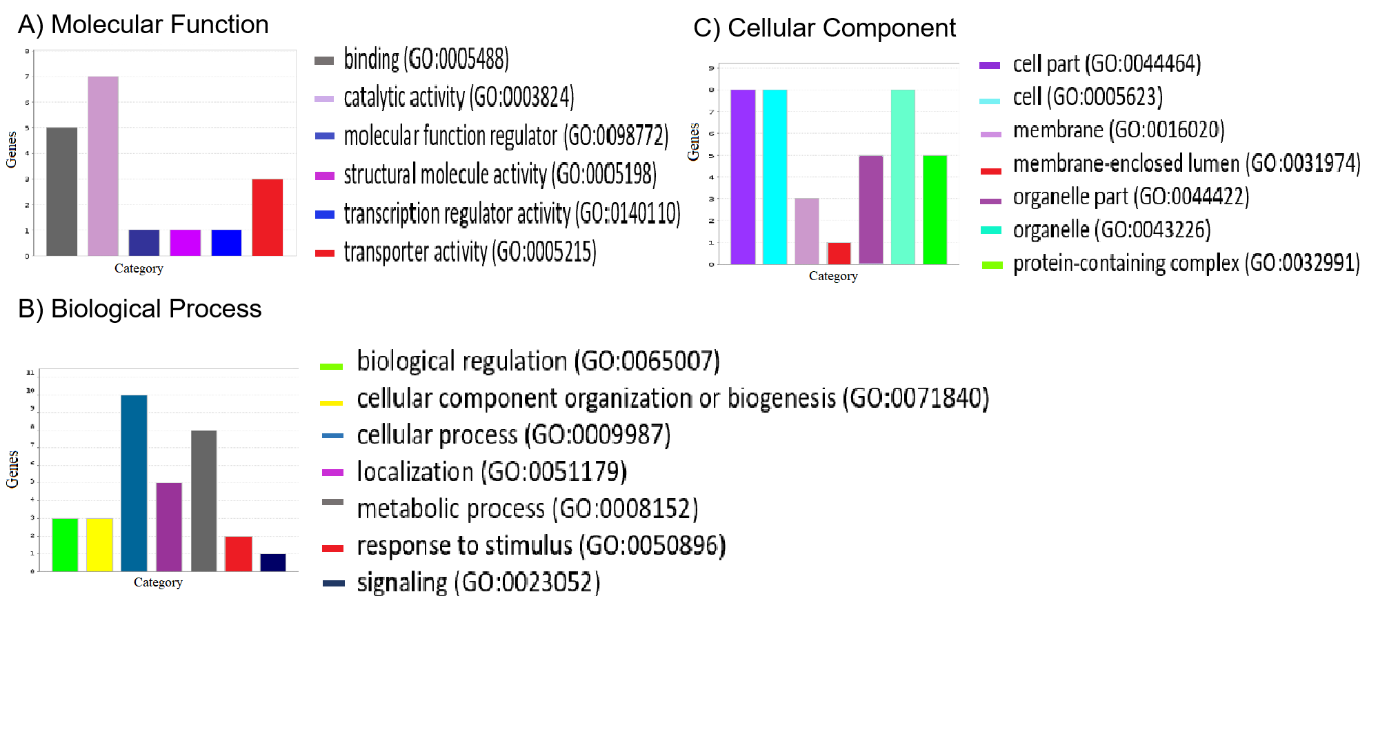


**FIGURE S2.** Go terms annotation for genes associated (p-value<0.05) with differentially expressed lncRNA in *Longissimus thoracis* of animals divergent for tenderness.


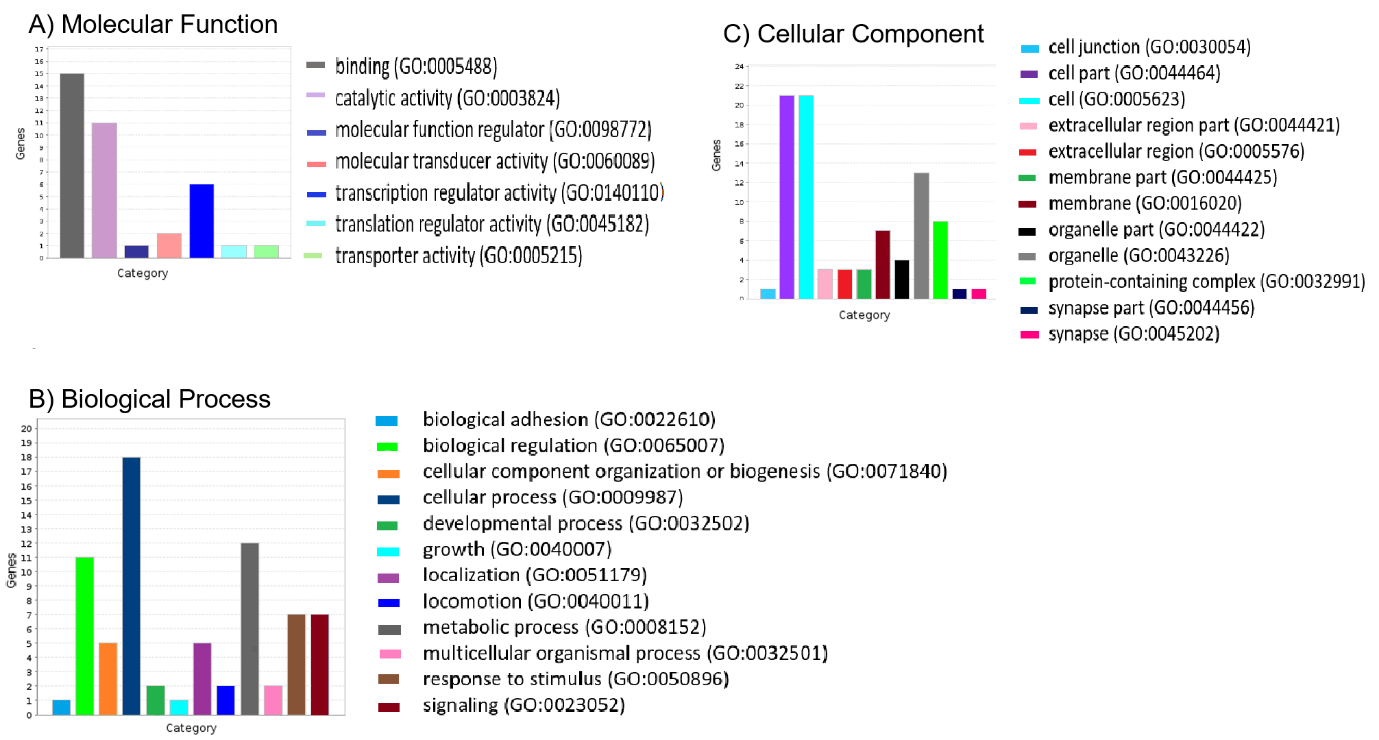


**FIGURE S3.** Go terms annotation for genes associated (p-value<0.05) with differentially expressed lncRNA in *Longissimus thoracis* of animals divergent for marbling.
